# The mediating role of behavioral risk factors in the pathway between childhood disadvantage and adult psychological distress in a Finnish employee cohort

**DOI:** 10.1038/s41598-024-74012-4

**Published:** 2024-10-08

**Authors:** Jatta Salmela, Tea Lallukka, Tomi Mäki-Opas, Luka Vähäsarja, Aino Salonsalmi

**Affiliations:** 1 Department of Public Health, University of Helsinki, Tukholmankatu 8 B, PO BOX 20, 00014 Helsinki, Finland; 2 Department of Social Sciences, University of Eastern Finland, Kuopio, Finland; 3 Wellbeing Services Research Centre, North Savo Wellbeing Services County, Kuopio, Finland

**Keywords:** Childhood disadvantage, Depression Anxiety Stress Scales 21, Health behaviors, Psychological distress, Structural equation modeling, Psychiatric disorders, Trauma, Psychology and behaviour, Socioeconomic scenarios

## Abstract

Childhood disadvantage is associated with increased adult psychological distress, but the role of behavioral risk factors in the pathway remains unclear. We examined whether behavioral risk factors mediate the effects of childhood disadvantage on adult psychological distress. We used the Helsinki Health Study data of employees of the City of Helsinki, Finland, aged 19–39 (mean age 32.0) years at baseline (2017). We included women (n = 2397) and men (n = 586) who responded to both baseline and follow-up (2022) surveys. At baseline, eight types of childhood disadvantage were asked retrospectively, and six adult behavioral risk factors were included. Psychological distress was measured by the DASS-21 at follow-up. We conducted mediation analyses using generalized structural equation modeling. Among women, we found indirect path effects of childhood disadvantage on adult psychological distress through behavioral risk factors (symptoms of depression: *β* = 0.68, 95% CI 0.20–1.17; anxiety: *β* = 0.54, 95% CI 0.13–0.95; and stress: *β* = 0.69, 95% CI 0.20–1.09). Among men, childhood disadvantage contributed only directly to adult depressive (*β* = 0.71, 95% CI 0.16–1.26) and stress (*β* = 0.61, 95% CI 0.10–1.13) symptoms. Our findings suggest that behavioral risk factors can mediate some of the adverse effects of childhood disadvantage on adult psychological distress among women.

## Introduction

Childhood disadvantage is a multidimensional concept that captures disadvantaged childhood social and socioeconomic circumstances and living conditions as well as adverse events and experiences, such as parental mental illness, parental divorce, family economic hardship, or being bullied by peers^1^. It is a well-known and common risk factor for adverse mental health outcomes in later life^1–3^. These outcomes include a wide spectrum of mental disorders, such as depressive and anxiety disorders, and post-traumatic stress disorder, as well as milder symptomatology, such as mild depression and stress overload^4,5^. Depressive and anxiety disorders are the most common mental disorders worldwide^6^, including Finland. While more than one in six (i.e., about 84 million) people across the EU countries was estimated to suffer from mental disorders in 2016—based on the data from the Institute for Health Metrics and Evaluation (IHME)—the estimated prevalence in Finland was every fifth people, being the highest rate among the EU countries^7^.

A comprehensive study, based on the World Mental Health Surveys from 21 countries, proposed that 29% of adult mental disorders in early adulthood (age 20–29) and 22% in later adulthood (age 30 +) were accounted for childhood adversities, measured by 12 types of adversities before the age of 18^8^. Additionally, a recent meta-analysis of 23 North American and European studies suggested that 31% of adult anxiety cases and 40% of adult depression cases in North America, and 28% of adult anxiety and depression cases in Europe, were attributed to childhood adversities^9^. Childhood adversities contributed more to anxiety and depression than to somatic diseases, such as cancer, cardiovascular disease, and diabetes, in terms of population-attributable fractions^9^. Given that the contribution of childhood disadvantage to adult mental health problems is substantial, it is crucial to understand the pathways in their interrelationship so that the adverse effects of childhood disadvantage on adult mental health can be minimized.

The life-course approach to health provides simplified conceptual models through which the impact of adverse early-life exposures on later health can be acknowledged, including (a) the ‘critical period models’ where the early-life risk factors are seen to permanently alter bodily functions, resulting in later diseases, and (b) the ‘accumulation of risk models’ where clustering, accumulation, and chaining of risk factors over time cumulatively increase later disease risk^10,11^. Childhood disadvantage can have both direct and indirect effects on adult mental health, being transmitted through dynamic biological, epigenetic, psychological, social, and behavioral pathways throughout the life-course^10,12^. For example, prolonged stress due to childhood adversities and its effects on bodily function (e.g., dysregulation of hormones, the immune system, and the functioning of the hypothalamic–pituitary–adrenal axis) are often stated in the literature to increase the risk for future mental health problems^13,14^. These biological changes are suggested to be embedded in individuals’ behavior (e.g., diet, exercise, and stress management), which further influence adult health outcomes^15^. Childhood disadvantage can also result in negative cognitions and beliefs of oneself, decreased perceived social support, and lower adult socioeconomic status, which further increase vulnerability to adult mental health problems^13,16^.

Although there are many proposed pathways on how childhood disadvantage can influence adult mental health, the mediators in the pathway need further examination^4,15,17^. Especially, understanding the mediating role of modifiable factors can provide insights on whether the adverse effects of childhood disadvantage on adult mental health can be diminished and intervened. Behavioral factors have been suggested to play a central role in how childhood disadvantage results in later health^15^, but only a few studies have specifically examined their role. Childhood disadvantage has been associated with several behavioral risk factors, such as problematic alcohol use, tobacco use, and obesity^2,18^, and these can further affect mental health^19–22^. Because behavioral risk factors tend to cluster and co-occur^23,24^, it is important to consider several behavioral risk factors together and simultaneously. Like behavioral risk factors, different types of childhood disadvantage tend to accumulate, especially among those with lower socioeconomic positions^15,25^. Both the quantity and type of childhood disadvantage influence the risk of adult mental health problems, which highlights the need for considering the multidimensionality of childhood disadvantage^26^. An exposure to multiple types of childhood disadvantage particularly increases the risk for adult mental health problems, supporting the ‘accumulation of risk models’^2,4,13,27^. However, certain types of childhood disadvantage, such as parental mental illness, violence at home, and bullying, seem to be more detrimental than others with respect to mental health^4,8,13^.

This study examines the mediating role of lifestyle- and health-related risk factors, which we more concisely refer to ‘behavioral risk factors’, in the pathway between childhood disadvantage and adult psychological distress among young and midlife Finnish employees. To the best of our knowledge, no previous studies have specifically examined the mediating role of behavioral risk factors in this pathway. We focus on depressive, anxiety, and stress symptoms as an outcome of psychopathological symptoms and use the general term ‘psychological distress’ to describe this outcome^28^. Mental disorders have become the main cause for sickness absence in Finland, and the steepest increase is seen among young and midlife women^29^. Thus, identifying the pathways that can lead to the development of mental health problems among working-aged Finns is particularly important so that employees’ psychological wellbeing and workability could be better supported in a long term. Previous studies suggest that the mechanisms between childhood disadvantage and adult psychological distress may differ between gender^30^, and behavioral risk factors are also modified by gender^24^, thus we examined women and men separately. While previous research on the topic is mostly based on traditional regression analyses, we utilized advanced statistical methods—that is, structural equation modeling (SEM)—to consider the multidimensionality of childhood disadvantage, behavioral risk factors, and psychological distress, and to assess the pathways between them.

Our research questions were:

- What are the direct, indirect, and total path effects of childhood disadvantage on adult psychological distress through adult behavioral risk factors?
- How different types of childhood disadvantage and adult behavioral risk factors contribute to the latent variables in these pathways?

## Methods

### Data and study participants

The data were derived from the Helsinki Health Study cohort surveys in 2017 (Phase 1) and 2022 (Phase 2). The Phase 1 questionnaires were sent to all employees of the City of Helsinki, Finland, who were born in 1978 or later, who had a job contract of at least 50% of regular working hours per week, and whose employment contract had lasted at least 4 months before the data collection began in autumn 2017 (N = 11,459)^31^. The surveys included a large set of questions on employees’ social-, work-, and health-related factors and behavioral factors. The data were collected via online and mailed surveys, and shorter telephone interviews were conducted to target those who did not otherwise respond. Participants could answer to the surveys in Finnish, Swedish, English, or Russian, and change the language at any time.

**Fig. 1:**
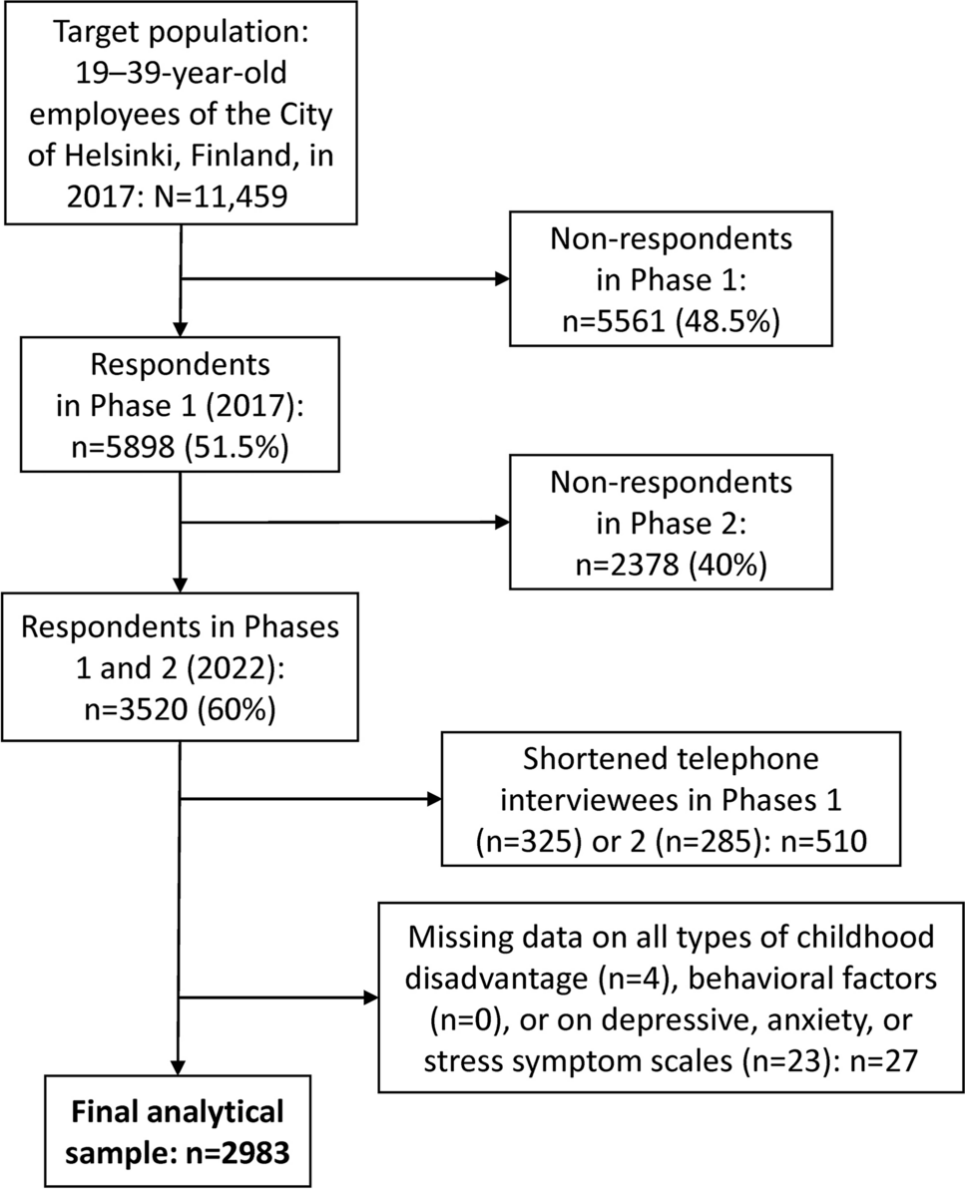
Flowchart of the selection of the Helsinki Health Study cohort participants in 2017–2022.

Those who responded in Phase 1 received the follow-up survey via online or mail in 2022. As in Phase 1, short telephone interviews were conducted among those who did not return the full follow-up survey. We only included participants who responded to both Phase 1 and 2 surveys (n = 3520). Telephone interviewees were excluded as the interviews did not comprise the variables of interest of this study. After further exclusions, the final sample included 2397 women and 586 men (Fig. 1). The gender distribution (80% women) corresponds to the general demographics of the employees of the City of Helsinki and the municipal sector in Finland^31^. The majority of the City of Helsinki employees, as the municipal employees in general in Finland, work in female-dominated occupations and sectors, such as social and healthcare services and education.

The Helsinki Health Study protocol has been approved by the ethics committees of the Faculty of Medicine, University of Helsinki, Finland, and the health authorities of the City of Helsinki, Finland. Appropriate ethical aspects have been followed in all phases of the study, according to the 1964 Helsinki Declaration. All participants were informed about the study and its purposes; voluntary participation; data confidentiality, use, and processing; data protection statement; and contact details. The participants did not receive any rewards in return of participation.

### Measures

#### Childhood disadvantage

We measured childhood disadvantage by eight indicators, consisting of seven childhood adversities and low parental education^32^. Participants were asked in Phase 1 if they experienced any of the following adversities before the age of 16 (yes/no): own serious or long-term illness; parental divorce, parental death, parental mental illness, and parental alcohol problems; family economic hardship; and being repeatedly bullied by peers. Paternal and maternal highest educational attainment were asked in Phase 1, and the highest one was considered for the combined measure. We dichotomized parental education into low (elementary or vocational school) and high (upper secondary school or university degree).

#### Behavioral risk factors

We derived adult behavioral factors from Phase 1, including six measures: fruit and vegetable (F&V) consumption, leisure-time physical activity (LTPA), alcohol use, tobacco use, sleep problems, and body mass index (BMI). Each measure was dichotomized so that they could separate those with risky or unhealthy behaviors from others, given that we hypothesized that behavioral risk factors could mediate the adverse effects of childhood disadvantage on adult psychological distress. We used ‘behavioral risk factors’ as an umbrella term for these lifestyle-related measures though we acknowledge that sleep problems and obesity are not, strictly speaking, behaviors and that not all behavioral risk factors (e.g., those related to personality) are lifestyle-related like our measures.

We derived F&V consumption from a 14-item food frequency questionnaire where participants estimated how often they usually consumed each food item during the past 4 weeks. We dichotomized F&V consumption into daily and non-daily consumption of fruit, berries, or fresh or cooked vegetables.

LTPA included the questions of participants’ weekly volume and intensity of exercise in leisure time or while commuting during the past 12 months. Four levels of intensity were provided, and they were multiplied by the time used per week in LTPA, yielding weekly metabolic equivalent task (MET)-hours^33,34^. We dichotomized LTPA into those with high or moderate (≥ 20 MET-hours/week) and those with low (< 20 MET-hours/week) LTPA^24^. Twenty MET-hours equals, for instance, 2.5 h brisk walking and 1.5 h walking, which closely correspond to the current guidelines^35,36^.

We derived alcohol use from the measures of total weekly alcohol use and binge drinking behavior. Participants estimated how often, on average, they consume different alcohol types: beer/cider, wine, and spirits. Seven frequency alternatives were provided for each question, with one unit of alcohol equaling 12 g of pure alcohol^37^. Additionally, participants were provided with six response alternatives (from ‘never’ to ‘daily/almost daily’) to estimate how often they drink six units or more of alcohol at once. We classified those drinking 0–7 (women) or 0–14 (men) units of alcohol per week—based on the Finnish Current Care Guidelines for moderate risk levels^37^—and binge drinking less than once a month into moderate alcohol users. Others were classified as excessive alcohol users.

Participants estimated their use of tobacco products (cigarettes, e-cigarettes, and snus) by four response alternatives. We dichotomized tobacco use into never/not anymore and occasionally/daily. Of those smoking occasionally/daily, 87% used cigarettes, 6% used e-cigarettes, and 15% (5% of women and 41% of men) used snus.

Sleep problems were measured by the Jenkins Sleep Questionnaire^38^. Participants were asked about how often during the past four weeks they (1) had trouble falling asleep, (2) woke up several times per night, (3) had trouble staying asleep, and (4) felt tired and worn out after waking up after a usual amount of sleep. Each of the 4 items included 6 response alternatives from ‘not at all’ to ‘22–28 nights’. We dichotomized participants into those having no/seldom and those having frequent sleep problems, using a cut point of 14 nights per 4 weeks^39^. BMI was calculated by dividing the self-reported weight (kg) by the self-reported height squared (m^2^). We dichotomized participants by using a cut point for obesity, that is, 30 kg/m^2^.

#### Psychological distress

We used the 21-item Depression Anxiety Stress Scales (DASS-21) questionnaire—with its different language translations^40–42^—from Phase 2 to measure adult psychological distress. The DASS-21 is widely used to measure negative emotional states and psychological distress among diverse population groups. In the DASS-21 questionnaire, participants estimated how the 21 statements applied to them over the past week with a 4-point scale (1–4 which was converted to 0–3) from ‘did not apply to me at all’ to ‘applied to me very much, or most of the time’. Subscales of depression, anxiety, and stress each included seven items (see Supplementary Information 1). The item scores were summed up in each subscale, higher scores indicating more frequent symptomatology. One missing item per scale was allowed when summing up the item scores^42^. Previous studies have reported good internal consistency reliability for each subscale, both in clinical and non-clinical samples^43–46^. While the subscales are greatly inter-correlated, they also contain variance that is specific to each subscale^40,44^. The depression scale mostly reflects dysphoric mood (e.g., hopelessness and lack of interest), anxiety scale assesses symptoms related to physical arousal, panic attacks, and fear, and stress scale measures symptoms such as tension, irritability, and over-reactivity to stressful events^40,43^. In each subscale, participants were classified conventionally as having normal, mild, moderate, and severe symptoms (see Table 1)^41^. We combined those reporting extremely severe symptoms with those reporting severe symptoms because there were few participants in these classes.

**Table 1:** Descriptive statistics of the Helsinki Health Study cohort participants (n, %) in the Phase 1 (2017) and 2 (2022) surveys.

	All participants (n = 2983)	Women (n = 2397)	Men (n = 586)	Chi^2^ test, *p*-value
Age (2017)				0.003
19–24	151 (5)	130 (5)	21 (4)	
25–29	694 (23)	572 (24)	122 (21)	
30–34	1010 (34)	826 (34)	184 (31)	
35–40	1128 (38)	869 (36)	259 (44)	
Childhood disadvantage (2017)				
Own serious/long-term illness				0.801
No	2678 (93)	2154 (93)	524 (93)	
Yes	207 (7)	165 (7)	42 (7)	
Parental divorce				0.176
No	2035 (70)	1649 (70)	386 (67)	
Yes	880 (30)	694 (30)	186 (33)	
Parental death				0.849
No	2710 (94)	2177 (94)	533 (94)	
Yes	173 (6)	140 (6)	33 (6)	
Parental mental illness				0.097
No	2449 (85)	1956 (84)	493 (87)	
Yes	448 (15)	373 (16)	75 (13)	
Parental alcohol problems				0.302
No	2120 (73)	1694 (72)	426 (74)	
Yes	794 (27)	648 (28)	146 (26)	
Family economic hardship				0.175
No	2275 (78)	1840 (79)	435 (76)	
Yes	636 (22)	499 (21)	137 (24)	
Peer bullied				0.177
No	2135 (73)	1728 (74)	407 (71)	
Yes	779 (27)	613 (26)	166 (29)	
Parental education				0.468
High	1674 (56)	1337 (56)	337 (56)	
Low	1306 (44)	1057 (44)	249 (42)	
Behavioral risk factors (2017)				
Fruit and vegetable consumption				< 0.001
Daily	2267 (76)	1907 (80)	360 (62)	
Non-daily	710 (24)	486 (20)	224 (38)	
Leisure-time physical activity				0.118
High/moderate	2492 (84)	1992 (84)	500 (87)	
Low	461 (16)	383 (16)	78 (13)	
Alcohol use				< 0.001
No/moderately	2200 (76)	1850 (80)	350 (61)	
Excessively	689 (24)	461 (20)	228 (39)	
Tobacco use				< 0.001
No/not nowadays	2206 (74)	1828 (77)	378 (65)	
Occasionally/daily	757 (26)	551 (23)	206 (35)	
Sleep problems				< 0.001
No/seldom	2048 (69)	1586 (66)	462 (79)	
Frequently	930 (31)	807 (34)	123 (21)	
Body mass index				0.520
< 30 kg/m^2^	2503 (85)	2014 (85)	489 (84)	
≥ 30 kg/m^2^	446 (15)	353 (15)	93 (16)	
Psychological distress (2022)				
Depressive symptoms (scores)				0.004
Normal (0–9)	2191 (73)	1793 (75)	398 (68)	
Mild (10–13)	281 (9)	209 (9)	72 (12)	
Moderate (14–20)	316 (11)	249 (10)	67 (11)	
Severe (21+)	195 (7)	146 (6)	49 (8)	
Anxiety symptoms (scores)				0.368
Normal (0–7)	2305 (77)	1840 (77)	465 (77)	
Mild (8–9)	195 (7)	162 (7)	33 (7)	
Moderate (10–14)	300 (10)	250 (10)	50 (10)	
Severe (15+)	183 (6)	145 (6)	38 (6)	
Stress symptoms (scores)				0.022
Normal (0–14)	2292 (77)	1814 (76)	478 (82)	
Mild (15–18)	279 (9)	239 (10)	40 (7)	
Moderate (19–25)	219 (7)	183 (8)	36 (6)	
Severe (26+)	193 (6)	161 (7)	32 (5)	

### Statistical methods

All analyses were conducted with Stata 18 (StataCorp LLC, College Station, TX, USA). We performed the analyses separately for women and men based on the literature^30^ and several statistically significant gender interactions found between the measures of childhood disadvantage and psychological distress, and the measures of behavioral risk factors and psychological distress. We first tabulated descriptive statistics (numbers, percentages, and *p*-values from the Pearson Chi^2^ tests) concerning all measures. Then, we examined the correlations between the measures of childhood disadvantage, behavioral risk factors, and the DASS-21 subscales with Spearman’s correlation. We also cross-tabulated the measures of childhood disadvantage and behavioral risk factors by the DASS-21 subscales.

**Fig. 2:**
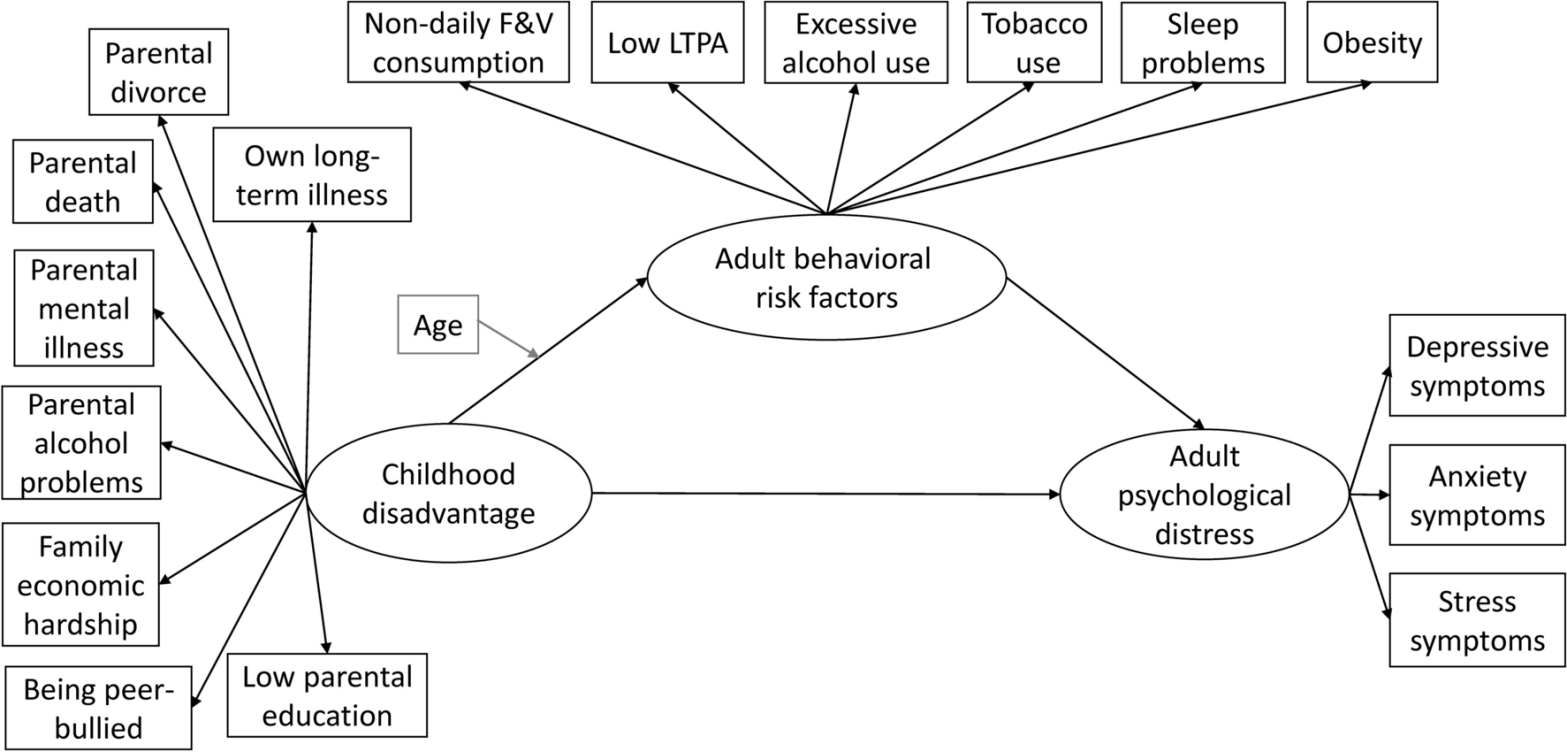
The conceptual pathway model to illustrate the pathways between childhood disadvantage, adult behavioral risk factors, and adult psychological distress. Circles, latent variables; boxes, observed variables. F&V, fruit and vegetable; LTPA, leisure-time physical activity.

To examine pathways between childhood disadvantage, adult behavioral risk factors, and adult psychological distress (Fig. 2), we used generalized structural equation modeling (GSEM) with Stata’s gsem command^47^. The distributions of continuous DASS-21 subscales were strongly right-skewed, thus we used GSEM which fits generalized linear response variables. We handled the childhood disadvantage and adult behavioral risk factor measures as binary variables (model: logit), the depression, anxiety, and stress subscales as ordinal (model: ologit), and the formed latent variables as continuous (model: linear regression). We used quasi-maximum likelihood to estimate standardized regression coefficients and to produce their robust standard errors and 95% confidence intervals (CIs). Quasi-maximum likelihood uses maximum likelihood to fit the model parameters but relaxes the normality assumptions when estimating the standard errors^47^.

We constituted latent variables for childhood disadvantage and adult behavioral risk factors by using model fit statistics to select which single measures were included in the latent variables. To compare two competitive GSEM models, we used the goodness-of-fit statistics that are available with Stata’s gsem command: Akaike Information Criteria and Bayesian Information Criteria (lower values indicating better fit), and likelihood-ratio tests (*p* < 0.05)^47^. Based on these model fit statistics, we removed tobacco use in women and sleep problems in men from the latent variables, and age was considered only in the pathway from childhood disadvantage to adult behavioral risk factors.

As a supplementary analysis, we constituted a latent variable for general psychological distress which combined the subscales of DASS-21 (Supplementary Information 2, Figs. S1–S4), given that the DASS-21 subscales also share a common factor of psychological distress^44,48^. The total path effects of childhood disadvantage on general psychological distress were greater than in the separate models for depressive, anxiety, and stress symptoms among women and men, and stress contributed most to the latent variable. However, these models did not achieve convergence until 300 iterations and the CIs were large, thus we omitted them from the main analyses, and these supplementary results should be interpreted with caution.

## Results

The most common types of childhood disadvantage were low parental education (44% of participants), parental divorce (30%), parental alcohol problems (27%), and being repeatedly bullied by peers (27%) (Table 1). There were no statistically significant gender differences in any type of childhood disadvantage, but gender differences were found in behavioral risk factors and psychological distress. Frequent sleep problems were more common among women than men (34% vs. 21%) whereas non-daily F&V consumption (20% vs. 38%), excessive alcohol use (20% vs. 39%), and tobacco use (23% vs. 35%) were less common among women. Approximately three in four participants had depressive, anxiety, and stress symptoms within ‘normal’ range. Women more often had depressive symptoms within the ‘normal’ range than men (75% vs. 68%) but more seldom stress symptoms within the ‘normal’ range (76% vs. 82%).

We found moderate correlations between the DASS-21 subscales (*β* = 0.48–0.62) among women and men (Supplementary Information 2, Table S1). Moderate correlations were also found between alcohol use and tobacco use (*β* = 0.33) and parental divorce and parental alcohol problems (*β* = 0.30) among women, and between parental divorce and parental alcohol problems (*β* = 0.35), parental alcohol problems and family economic hardship (*β* = 0.35), and parental divorce and family economic hardship (*β* = 0.32) among men. Having depressive, anxiety, and stress symptoms within the ‘normal’ range was less common among women who had experienced childhood disadvantage, except low parental education and parental death, and with excessive alcohol use, frequent sleep problems, and obesity (Supplementary Information 2, Table S2). Among men, having depressive, anxiety, and stress symptoms within the ‘normal’ range was less common among men who had experienced family economic hardship and peer bullying, and with low LTPA and frequent sleep problems (Supplementary Information 2, Table S3).

**Fig. 3:**
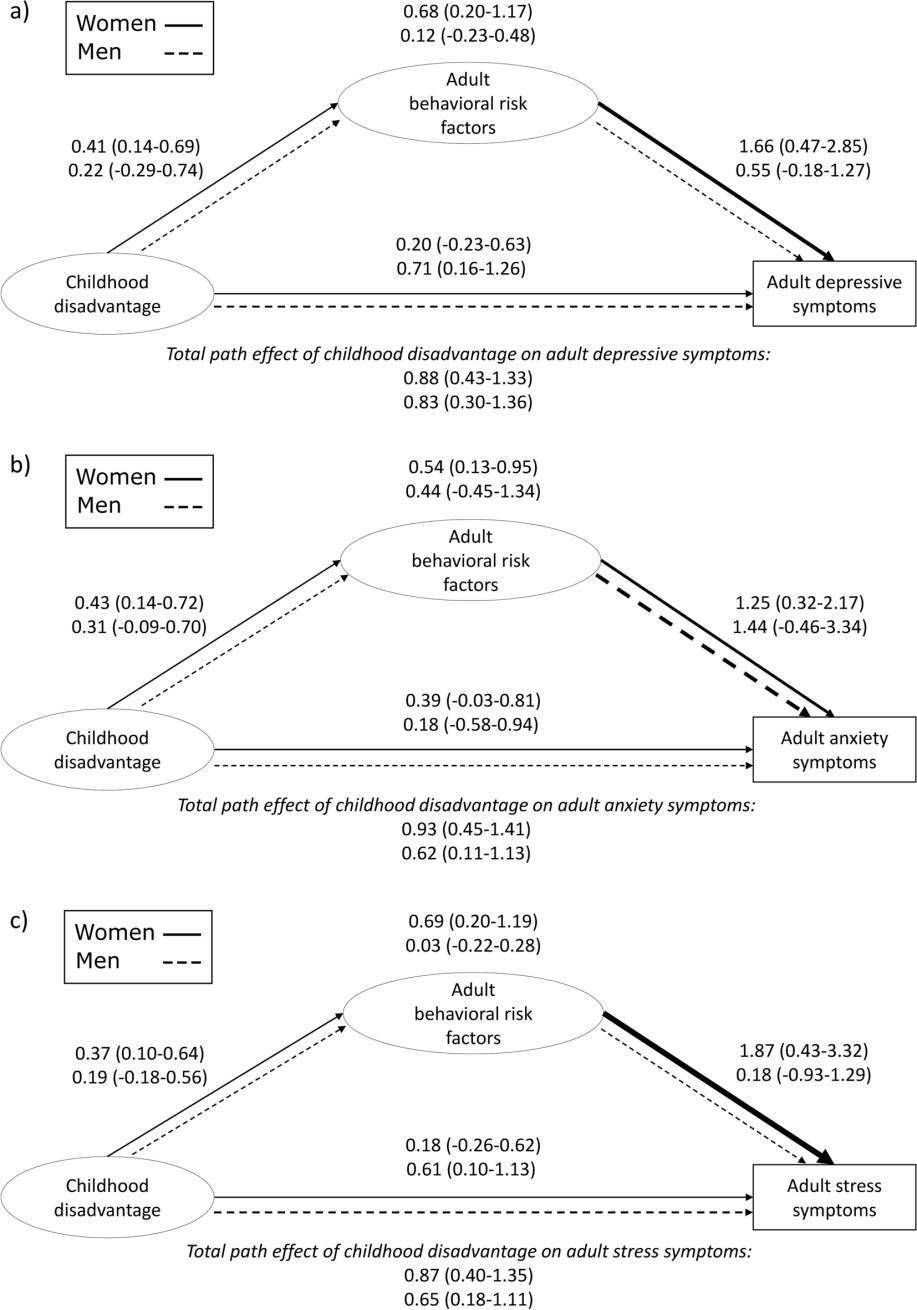
Generalized structural equation model for the pathways between childhood disadvantage (8 items), adult behavioral risk factors (5 items), and adult psychological distress using the subscales of Depression Anxiety Stress Scales 21 (DASS-21): (**a**) depressive, (**b**) anxiety, and (**c**) stress symptoms. The Helsinki Health Study cohort participants in Phases 1 (2017) and 2 (2022). The pathway effect of childhood disadvantage on adult behavioral risk factors is adjusted for age. Estimated path coefficients (*β*-values) and their 95% confidence intervals are shown: upper estimates and solid lines for women (n = 2397) and lower estimates and dashed lines for men (n = 586). Thicker lines indicate stronger path effects. Circles, latent variables; boxes, observed variables.

Among women, we found statistically significant indirect path effects of childhood disadvantage on adult depressive (*β* = 0.68, 95% CI 0.20–1.17), anxiety (*β* = 0.54, 95% CI 0.13–0.95), and stress (*β* = 0.69, 95% CI 0.20–1.19) symptoms through adult behavioral risk factors, and these path effects constituted most of the total path effects between childhood disadvantage and psychological distress (Fig. 3). Additionally, we found statistically significant direct path effects of childhood disadvantage on behavioral risk factors and, to a greater extent, of behavioral risk factors on psychological distress. The direct path effects of childhood disadvantage on psychological distress were modest and statistically insignificant. Parental divorce, mental illness, and alcohol problems, and family economic hardship contributed most to the latent childhood disadvantage variable, and frequent sleep problems and obesity to the latent behavioral risk factor variable among women (Supplementary Information 2, Figs. S5–S6).

Among men, we did not find indirect path effects of childhood disadvantage on adult psychological distress through adult behavioral risk factors (Fig. 3). However, there were statistically significant direct path effects of childhood disadvantage on depressive symptoms (*β* = 0.71, 95% CI 0.16–1.26) and stress symptoms (*β* = 0.61, 95% CI 0.10–1.13). Parental alcohol problems and family economic hardship contributed most to the latent childhood disadvantage variable, and tobacco use to the latent behavioral risk factor variable among men (Supplementary Information 2, Figure S5–S6).

## Discussion

In this cohort study of young and midlife Finnish employees, we found that behavioral risk factors partly mediated the associations between childhood disadvantage and adult psychological distress among women but not among men. Among women, we did not find any statistically significant direct path effects between childhood disadvantage and psychological distress as we found for men (concerning depressive and stress symptoms). Instead, adult behavioral risk factors had direct path effects to psychological distress among women but not among men. A novelty of our study was that we could consider several types of childhood disadvantage and adult behavioral risk factors and examine their contributions to the analyses. The most influential childhood disadvantage measures were family economic hardship and parental alcohol problems, and among women, sleep problems and obesity contributed most to the latent variable of adult behavioral risk factors.

Experiencing childhood disadvantage was common among the study participants, of which 78% had experienced any type of childhood disadvantage. Even after excluding low parental education, which was relatively common, still 65% of women and 66% of men reported any childhood adversities. Another Finnish study of working-aged adults found that 45% of women and 39% of men reported any childhood adversities^49^, and in a large World Mental Health Survey, the share was 38% among participants from high-income countries^8^. Our shares were also higher than among the older Helsinki Health Study cohort of 40–60-year-old employees of the City of Helsinki, Finland, where approximately half of the participants reported any childhood adversities^32^. Especially, parental divorce, mental illness, and alcohol problems, and being peer bullied were more common in this younger cohort. Some of these cohort differences probably illustrate true changes in the prevalence of childhood adversities (e.g., increased rates of divorces and binge drinking^50,51^), while some of them are probably related to recall and selection biases in reporting (e.g., concerning parental mental illness)^52^. Since all participants were municipal employees in Phase 1, they do not represent the most marginalized groups (e.g., people outside the labor force) in our society, among whom childhood adversities could be even more common.

Behavioral risk factors operated as a mediator between childhood disadvantage and adult psychological distress among women but not men. A few previous studies have examined the mediating role of behavioral risk factors in the association of childhood disadvantage with adult mental health and have found mostly partial mediation. A representative Norwegian cohort study of adults living in Tromsø found that smoking and alcohol use together with low social support mediated 15% (95% CI 8.94–27.16) of the associations between childhood traumatic experiences and poor adult mental health^53^. A longitudinal Canadian study among university students found that clinically significant insomnia—but not nicotine, cannabis, and caffeine use, binge drinking, exercise, and BMI—partially mediated the associations between childhood abuse and mental health outcomes (i.e., clinically significant symptoms of depression and anxiety, suicidality, and self-harm)^54^. A cross-sectional American study of adults living in a low-income community in Florida examined the mediating effects of several health behaviors (i.e., smoking, alcohol use, diet, physical activity, and sleep disturbances) on the relationship between childhood adversities and poor adult health-related quality of life (including physical and mental health), and found only sleep disturbances significantly mediating the effects (*β* = 0.11, 95% CI 0.03–0.21)^55^. None of these three studies examined women and men separately but used gender as a confounding variable in the mediation analyses.

The found mediating effects of behavioral risk factors among women support the ‘chains of risk model’ where one adverse exposure tends to lead to another and then another^10^. Among men, however, the ‘critical period model’^10^ seems to be more supported in our data, given that childhood had only direct effects on depressive and stress symptoms. Previous studies have pondered that the mediating role of behavioral risk factors between childhood disadvantage and adult mental health problems may imply that unhealthy behaviors operate as a coping mechanism to handle stressful childhood conditions^53,56^. On the contrary, adaptation to healthy behaviors after disadvantaged childhood may indicate higher levels of resilience and further better psychological well-being^57^. It is also notable that behavioral risk factors do not operate separately from other factors in the pathway, such as psychosocial, biological, and cognitive factors (e.g., stress, self-esteem, social support, and coping strategies) and individual’s own socioeconomic position^30,53,54,57^. Additionally, the mediation mechanisms may differ between genders—for instance, due to different coping mechanisms in handling stressful circumstances—^30,58^, suggested also by our findings. Given that the study settings (cross-sectional, prospective, retrospective) and the measures used widely vary between studies^30,53–55^, the comparisons are challenging. Nevertheless, the pathways from childhood disadvantage were somewhat similar between depressive, anxiety, and stress outcomes among women, which suggests that childhood disadvantage may have generic rather than specific effects on adult psychological distress. This is in line with previous research, although the possible specificity in the pathways should not be ignored^59^.

Although the conceptual pathway model illustrates causal relationships from childhood disadvantage to adult psychological distress through behavioral risk factors, the reverse causalities are also possible. A child’s psychological characteristics, such as social anxiety and certain personality traits, can influence risk taking behavior and traumatic events in adolescence^60–62^, which can further influence later mental health. The first onset of mental disorders often occurs by young adulthood^63^, which may be caused by both the genetic heritability of mental health problems and the long-term impact of adverse childhood environment on mental health^60^. However, in depressive disorder, for example, the first onset can occur even closer to middle age or older^63^. The heritability of mental health problems has been suggested to be lower among those having lived with childhood disadvantage, and childhood disadvantage might moderate the heritability of mental health problems^60,64^. Additionally, social causation hypothesis is supported over social selection in the framework of childhood socioeconomic circumstances and mental health, highlighting the detrimental impact of childhood disadvantage on later mental health^65^. Moreover, not only parental mental illness but also parental alcohol problems, parental divorce, and family economic hardship clearly contributed to the latent variable of childhood disadvantage in our pathway models, suggesting that the heritability of psychological distress is probably not the key explanatory factor in the effects found. Finally, behavioral risk factors (e.g., obesity and sleep problems) may partly result from psychological distress, not only the other way around.

The strength of this study lies in the comprehensive survey data of the City of Helsinki employee cohort, with a variety of questions on participants’ social- and health-related factors, from present and past. The DASS-21 measure we used has recently been validated in Finnish (unpublished paper under review). The follow-up setting strengthened the conceptual basis for conducting mediation analyses, which often rely on cross-sectional data^30^. Additionally, no previous studies, to the best of our knowledge, have used SEM approach with latent variables to investigate how behavioral factors mediate the pathways from childhood disadvantage to adult psychological distress. By using latent variables, we did not only consider the quantity of different exposures but also their mutual importance in the pathways, which is important since both quantity, type, and severity of childhood disadvantage is known to influence mental health^26,30^. Previous studies suggest that psychopathological symptoms, such as psychological distress, mediate themselves the pathway from childhood disadvantage to more serious mental disorders^30,66,67^. Thus, our study produces important knowledge from the preventive perspective—with respect to developing serious mental disorders and its impact on workability—given that our focus was on the milder symptoms of mental health problems and the participants were young and midlife employees at their early career.

The limitations of this study should be considered. Firstly, our study setting and mediation analyses do not enable us to verify causal relationships, and the ‘effects’ are statistical rather than true causal effects^68^. Secondly, since our data are based on self-reports and retrospective reports on childhood disadvantage, reporting and recall biases cannot be ruled out^52,69^. Socially desirable behaviors, such as F&V consumption and LTPA, are often overestimated^69^. Individuals with psychological distress are more likely to report childhood adversities^70^, thus the associations between childhood disadvantage and adult psychological distress may be overestimated. However, false positive reports are likely rare, especially concerning serious adversities^71^. Thirdly, the number of men (n = 586) was much smaller than that of women (n = 2397), which weakens between-gender comparisons, and the statistically insignificant findings among men may reflect lack of statistical power.

Finally, missing data, follow-up attrition and the fact that the participants were all municipal employees in Phase 1 may have caused social- and health-related selection to the sample. For example, while 86% of those responding to the questions on childhood disadvantage in Phase 1 reported any childhood disadvantage, the corresponding share among the Phase 2 respondents was 78%. In addition, non-daily F&V consumption, excessive alcohol use, and tobacco use were slightly less common in the final study sample (n = 2983) compared to all Phase 1 respondents (n = 5898). Although the Phase 1 respondents broadly represented the target population, women and the socioeconomically more advantaged were slightly overrepresented compared to the target population^31^. Overall, the participants in the final study sample were likely healthier and socially more advantaged than the target population, 19–39 employees of the City of Helsinki.

In conclusion, this study gives insight on how behavioral risk factors might mediate the effects of childhood disadvantage on adult psychological distress among an employee cohort. Interventional studies are needed to examine whether promoting healthy behaviors among individuals with childhood disadvantage could improve their mental health. These individuals might need special support (e.g., from mental health specialists) for the adherence of healthy behaviors. Future studies could also examine how the timing (e.g., in childhood or in adulthood) of adherence to healthy behaviors would impact on how behavioral factors mediate the effects of childhood disadvantage on adult mental health, and whether it is modified by gender. Nevertheless, early intervening and identifying the most vulnerable population groups is crucial, and awareness of the adverse effects of childhood disadvantage on adult mental health should be increased in healthcare and in societies more broadly.

## Supplementary Information

Supplementary Information 1.

Supplementary Information 2.

## Data Availability

The Helsinki Health Study survey data cannot be made publicly available due to strict data protection laws and regulations. The data can only be used for scientific research. More information on the survey data can be requested from the Helsinki Health Study research group (kttl-hhs@helsinki.fi).

## Abbreviations

BMI: Body mass index
CI: Confidence interval
DASS-21: 21-Item Depression Anxiety Stress Scales
F&V: Fruit and vegetable
GSEM: Generalized structural equation modeling
LTPA: Leisure-time physical activity
MET: Metabolic equivalent task
SEM: Structural equation modeling
