# Supplementary material for: The mediating role of behavioral risk factors in the pathway between childhood disadvantage and adult psychological distress in a Finnish employee cohort
Source: Sci Rep. 2024 Oct 8;14:23422. doi: 10.1038/s41598-024-74012-4 (PMC11461862; doi:10.1038/s41598-024-74012-4)
Supplement: Supplementary file 1 — Supplementary Information 1. [file 41598_2024_74012_MOESM1_ESM.pdf]

Supplementary Information 1

The relevant questions to this study from the Helsinki Health Study questionnaire surveys from Phases 1 (2017) and 2 (2022)

Phase 1, 2017

7. What is/was your mother's highest level of education?

- ❑ Elementary school, primary school, middle school or less
- ❑ Vocational school, college or equivalent
- ❑ Upper secondary degree/qualification/university degree

8. What is/was your mother's highest level of education?

- ❑ Elementary school, primary school, middle school or less
- ❑ Vocational school, college or equivalent
- ❑ Upper secondary degree/qualification/university degree

13. Did any of the following happen during your childhood (that is, up until you were 16)?

- ❑ You were seriously or chronically ill
- ❑ Your parents were divorced
- ❑ One or both of your parents died
- ❑ Your mother or father had mental health issues, which caused difficulties at home
- ❑ Your mother's or father's consumption of alcohol caused problems at home
- ❑ Your family had major financial difficulties
- ❑ You were a victim of repeated bullying at school or among your peers

28 a. Do you smoke cigarettes?

- ❑ Yes, every day, how many cigarettes a day?______________________________
- ❑ Occasionally
- ❑ Not anymore - I quit smoking in (year)__________________________________
- ❑ I have never smoked

28 b. Do you use snuff?

- ❑ Yes, every day
- ❑ Occasionally
- ❑ Not anymore
- ❑ I have never used snuff

28 c. Do you use electronic cigarettes (vape)?

- ❑ Yes, every day
- ❑ Occasionally
- ❑ Not anymore
- ❑ I have never used an electronic cigarette

31. How much, on average, do you consume the following alcoholic beverage?

a. Beer or cider

- ❑ None
- ❑ Less than one bottle a week
- ❑ 1–4 bottles a week
- ❑ 5–12 bottles a week
- ❑ 13–24 bottles a week
- ❑ 25–47 bottles a week

- ❑ 48 bottles or more a week

b. Wine or equivalent alcoholic beverage

- ❑ None
- ❑ Less than a glass a week
- ❑ 1–4 glasses a week
- ❑ 1–2.5 bottles a week
- ❑ 3–4.5 bottles a week
- ❑ 5–9 bottles a week
- ❑ 10 bottles or more a week

c. Spirits

- ❑ Not at all
- ❑ Less than half a bottle a month
- ❑ 0.5–1.5 bottles a month
- ❑ 2–3.5 bottles a month
- ❑ 4–9 bottles a month
- ❑ 10–19 bottles a month
- ❑ 20 bottles or more a month

32. The next question concerns situations in which you drink six or more servings of alcoholic beverages at one sitting. Six or more servings is equivalent to at least:

- 4 pints (0.5 l each) medium-strength beer/mild cider or

- 3 pints (0.5 l each) strong beer/strong cider or- one bottle (0.75 l) of mild wine (12%) or

- 6 restaurant servings (4 cl each) of spirits

How often do you drink six or more servings of alcoholic beverages at one sitting?

- ❑ Never
- ❑ Less than once a month
- ❑ Once a month
- ❑ Once a week
- ❑ A few times a week
- ❑ Every day or almost every day

39. How often do you consume the following food items? Think about the past four weeks. Please choose one alternative in each line.

	Not in the past 4 weeks	1–3 times a month	Once a week	2–4 times a week	5–6 times a week	Once a day	2 times or more a day
Fresh vegetables or green salad							
Boiled vegetables							
Fruit							
Berries							

41. Next, we will be asking about physical activity during your leisure and commuting time over the past 12 months. We have divided physical activities in four levels of exertion. First, estimate the exertion level of the physical activities you are engaged in. Then, estimate how often you engage in a physical activity equivalent to each level of exertion during one week rounded to closest 15 minutes (e.g. 02 hours and 45 minutes).

a. During your leisure time

Strenuousness of exercise:	Hours	Minutes
Equivalent to walking		
Equivalent to brisk walking		
Equivalent to light running (jogging)		
Equivalent to brisk running		

b. During your commute

Strenuousness of exercise:	Hours	Minutes
Equivalent to walking		
Equivalent to brisk walking		
Equivalent to light running (jogging)		
Equivalent to brisk running		

43. How tall are you?

________________________________cm

44. How much do you weigh?

________________________________kg (round to the nearest kilogramme)

53. How often have you had the following symptoms during the past four weeks?

	Not at all	1–3 nights	4–7 nights	8–14 nights	15–21 nights	22–28 nights
Having trouble falling asleep						
Waking up several times per night						
Having trouble staying asleep (including waking up far too early)						
Feeling tired and worn out when waking up after the usual amount of sleep						

Phase 2, 2022

66. Please read each statement and circle a number 1, 2, 3 or 4 which indicates how much the statement applied to you over the past week. There are no right or wrong answers. Do not spend too much time on any statement.

The rating scale is as follows:

1 Did not apply to me at all

2 Applied to me to some degree, or some of the time

3 Applied to me to a considerable degree or a good part of time

4 Applied to me very much or most of the time

	1	2	3	4
a) I found it hard to wind down				
b) I was aware of dryness of my mouth				
c) I couldn’t seem to experience any positive feeling at all				
d) I experienced breathing difficulty (e.g. excessively rapid breathing, breathlessness in the absence of physical exertion)				
e) I found it difficult to work up the initiative to do things				
f) I tended to over-react to situations				
g) I experienced trembling (e.g. in the hands)				
h) I felt that I was using a lot of nervous energy				
i) I was worried about situations in which I might panic and make a fool of myself				
j) I felt that I had nothing to look forward to				
k) I found myself getting agitated				
l) I found it difficult to relax				
m) I felt down-hearted and blue				
n) I was intolerant of anything that kept me from getting on with what I was doing				
o) I felt I was close to panic				
p) I was unable to become enthusiastic about anything				
q) I felt I wasn’t worth much as a person				
r) I felt that I was rather touchy				
s) I was aware of the action of my heart in the absence of physical exertion (e.g. sense of heart rate increase, heart missing a beat)				
t) I felt scared without any good reason				
u) I felt that life was meaningless				

**Authors' note:* depression, anxiety, and stress scores were calculated based on the following questions: depression: c, e, j, m, p, q, u; anxiety: b, d, g, i, o, s, t; and stress: a, f, h, k, l, n, r*
