# Supplementary material for: The mediating role of behavioral risk factors in the pathway between childhood disadvantage and adult psychological distress in a Finnish employee cohort
Source: Sci Rep. 2024 Oct 8;14:23422. doi: 10.1038/s41598-024-74012-4 (PMC11461862; doi:10.1038/s41598-024-74012-4)
Supplement: Supplementary file 2 — Supplementary Information 2. [file 41598_2024_74012_MOESM2_ESM.pdf]

Supplementary Information 2

Table S1. Correlation matrix between the examined variables using Spearman's correlation test. Correlation coefficients and their statistical significance are shown for women (below the diagonal) and men (above the diagonal).

	**Own serious/ long-term illness**	**Parental divorce**	**Parental death**	**Parental mental illness**	**Parental alcohol problems**	**Family economic hardship**	**Peer-bullied**	**Parental education**	**Fruit and vegetable consumption**	**Leisure-time physical activity**	**Alcohol use**	**Tobacco use**	**Sleep problems**	**Body mass index**	**Depression**	**Anxiety**	**Stress**
**Own serious /long-term illness**	**1.00**	0.03	0.08	0.03	0.07	0.16***	0.16***	0.07	-0.02	0.04	0.05	0.05	0.06	0.10*	0.11*	0.10*	0.09*
**Parental divorce**	0.01	**1.00**	0.16***	0.19***	0.35***	0.32***	0.07	0.16***	0.06	-0.01	-0.02	0.10*	0.04	0.04	0.11*	0.08	0.03
**Parental death**	0.03	0.07**	**1.00**	0.11*	0.18***	0.12*	0.00	0.08	-0.01	0.00	-0.05	-0.03	0.05	-0.07	-0.02	-0.01	-0.04
**Parental mental illness**	0.09***	0.19***	0.04	**1.00**	0.23***	0.28***	0.22***	0.03	0.05	0.03	-0.02	-0.01	0.12**	0.03	0.10*	0.06	0.07
**Parental alcohol problems**	0.01	0.30***	0.10***	0.25***	**1.00**	0.35***	0.09*	0.12**	0.11*	-0.02	0.02	0.01	0.08	0.04	0.10*	0.02	0.05
**Family economic hardship**	0.02	0.24***	0.05*	0.25***	0.24***	**1.00**	0.18***	0.23***	0.08	0.11*	0.03	0.07	0.12**	0.08	0.22***	0.09	0.14**
**Peer bullied**	0.13***	0.02	0.04	0.12***	0.06**	0.18***	**1.00**	0.03	0.07	0.08	-0.01	0.03	0.16***	0.15	0.20***	0.17***	0.23***
**Parental education**	-0.01	0.08***	0.08***	0.00	0.14***	0.12***	0.06**	**1.00**	0.15***	0.07	-0.02	0.10*	0.07	0.07	0.08	0.05	0.07
**Fruit and vegetable consumption**	0.04	0.04*	0.01	0.02	0.03	0.06**	0.06**	0.07**	**1.00**	0.14**	0.03	0.11*	-0.03	0.07	0.07	0.09*	0.04
**Leisure-time physical activity**	-0.01	0.06**	0.02	-0.01	0.03	0.07**	0.05*	0.08***	0.11***	**1.00**	0.04	0.05	0.15***	0.08	0.17***	0.22***	0.23***
**Alcohol use**	0.01	0.03	0.01	0.02	0.04	-0.01	0.01	-0.01	0.07*	-0.01	**1.00**	0.27***	0.05	0.04	0.05	0.09*	0.00
**Tobacco use**	0.03	0.10***	0.04	0.06**	0.08***	0.08***	0.01*	0.05*	0.09***	0.01	0.33***	**1.00**	-0.06	0.05	0.09*	0.11*	0.03
**Sleep problems**	0.07**	0.03	0.00	0.07**	0.04*	0.06**	0.10***	0.00	0.03	0.08***	0.04*	0.01	**1.00**	0.09*	0.24***	0.17***	0.22***
**Body mass index**	0.03	0.03	0.01	0.04*	0.02	0.09***	0.19***	0.09***	0.07**	0.13***	0.03	0.07**	0.10***	**1.00**	0.11*	0.13**	0.08
**Depression**	0.06	0.07**	0.02	0.09***	0.08***	0.05*	0.12***	0.00	0.06**	0.03	0.10***	0.07**	0.19***	0.08***	**1.00**	0.55***	0.62***
**Anxiety**	0.07**	0.07***	0.03	0.10***	0.07***	0.08***	0.15***	0.03	0.05*	0.04	0.09***	0.08***	0.15***	0.06**	0.48***	**1.00**	0.59***
**Stress**	0.06**	0.06**	0.01	0.10***	0.05*	0.08***	0.12***	0.01	0.03	0.01	0.08***	0.06**	0.20***	0.07***	0.56***	0.53***	**1.00**

*$p \leq 0.05$, **$p \leq 0.01$, ***$p \leq 0.001$

Table S2. Descriptive statistics of childhood disadvantage and adult behavioral risk factors by adult psychological distress, measured by the subscales of the Depression Anxiety Stress Scales 21 (DASS-21), among women (n, %).

	Depressive symptoms					Anxiety symptoms					Stress symptoms				
Childhood disadvantage	**Normal**	**Mild**	**Moderate**	**Severe**	***p*** [a]	**Normal**	**Mild**	**Moderate**	**Severe**	***p*** [a]	**Normal**	**Mild**	**Moderate**	**Severe**	***p*** [a]
**Own serious/ long-term illness**					***					***					**
No	1632 (94)	189 (94)	208 (88)	125 (89)		1674 (94)	134 (88)	225 (93)	121 (87)		1649 (94)	204 (88)	166 (94)	135 (89)	
Yes	108 (6)	12 (6)	29 (12)	16 (11)		110 (6)	19 (12)	18 (7)	18 (13)		109 (6)	28 (12)	11 (6)	17 (11)	
**Parental divorce**					**					**					*
No	1267 (72)	138 (67)	148 (61)	96 (68)		1299 (72)	102 (65)	157 (65)	91 (64)		1274 (72)	161 (69)	118 (66)	96 (62)	
Yes	488 (28)	67 (33)	94 (39)	45 (32)		502 (28)	55 (35)	86 (35)	51 (36)		503 (28)	73 (31)	60 (34)	58 (38)	
**Parental death**															*
No	1641 (94)	188 (95)	219 (93)	129 (93)		1683 (94)	138 (92)	226 (94)	130 (92)		1651 (94)	219 (95)	172 (97)	135 (89)	
Yes	104 (6)	10 (5)	17 (7)	9 (7)		102 (6)	12 (8)	15 (6)	11 (8)		108 (6)	11 (5)	5 (3)	16 (11)	
**Parental mental illness**					***					***					***
No	1504 (86)	166 (82)	180 (76)	106 (75)		1539 (86)	122 (80)	200 (83)	95 (66)		1518 (86)	185 (79)	144 (81)	109 (70)	
Yes	243 (14)	37 (18)	58 (24)	35 (25)		252 (14)	31 (20)	41 (17)	49 (34)		244 (14)	49 (21)	34 (19)	46 (30)	
**Parental alcohol problems**					***					***					
No	1304 (74)	137 (67)	154 (63)	99 (71)		1335 (74)	102 (65)	169 (70)	88 (62)		1302 (74)	167 (71)	124 (69)	101 (65)	
Yes	449 (26)	68 (34)	90 (37)	41 (29)		465 (26)	54 (35)	74 (30)	55 (38)		469 (26)	69 (29)	56 (31)	54 (35)	
**Family economic hardship**					**					**					***
No	1402 (80)	164 (80)	171 (72)	103 (72)		1443 (80)	113 (73)	183 (75)	101 (71)		1423 (80)	176 (76)	138 (77)	103 (66)	
Yes	353 (20)	40 (20)	66 (28)	40 (28)		356 (20)	41 (27)	60 (25)	42 (29)		349 (20)	55 (24)	42 (23)	53 (34)	
**Peer bullied**					***					***					***
No	1354 (77)	131 (64)	161 (69)	82 (57)		1391 (77)	92 (59)	158 (65)	87 (61)		1356 (77)	163 (70)	122 (67)	87 (56)	
Yes	405 (23)	73 (36)	74 (31)	61 (43)		408 (23)	63 (41)	86 (35)	56 (39)		415 (23)	70 (30)	59 (33)	69 (44)	
**Parental education**															
High	997 (56)	128 (61)	132 (53)	80 (55)		1039 (57)	84 (52)	137 (55)	77 (53)		1016 (56)	131 (55)	106 (58)	84 (52)	
Low	794 (44)	81 (39)	116 (47)	66 (45)		799 (43)	78 (48)	113 (45)	67 (47)		796 (44)	107 (45)	77 (42)	77 (48)	
Behavioral factors															
**Fruit and vegetable consumption**					**										*
Daily	1446 (81)	173 (83)	185 (74)	103 (71)		1480 (81)	126 (78)	198 (80)	103 (72)		1455 (80)	196 (82)	142 (78)	114 (71)	
Non-daily	344 (19)	36 (17)	64 (26)	42 (29)		358 (19)	36 (22)	51 (20)	41 (28)		356 (20)	43 (18)	41 (22)	46 (29)	
**Leisure-time physical activity**										*					
High/moderate	1506 (85)	178 (86)	192 (79)	116 (81)		1546 (85)	134 (83)	190 (78)	122 (85)		1515 (84)	207 (87)	144 (80)	126 (81)	
Low	274 (15)	30 (14)	52 (21)	27 (19)		279 (15)	27 (17)	55 (22)	22 (15)		285 (16)	31 (13)	37 (20)	30 (19)	
**Alcohol use**					***					***					***
No/moderately	1418 (82)	160 (78)	177 (73)	95 (69)		1450 (82)	127 (79)	166 (69)	107 (75)		1425 (82)	188 (80)	129 (72)	108 (70)	

Excessively	308 (18)	46 (22)	64 (27)	43 (31)		317 (18)	33 (21)	76 (31)	35 (25)		318 (18)	48 (20)	49 (28)	46 (30)	
**Tobacco use**					***					***					***
No/not nowadays	1394 (78)	168 (81)	170 (69)	96 (66)		1437 (79)	123 (76)	179 (72)	89 (62)		1408 (78)	185 (78)	129 (71)	106 (66)	
Occasionally/daily	387 (22)	40 (19)	75 (31)	49 (34)		390 (21)	38 (24)	68 (28)	55 (38)		393 (22)	51 (22)	53 (29)	54 (34)	
**Sleep problems**					***					***					***
No/seldom	1267 (71)	137 (66)	120 (48)	62 (43)		1287 (70)	102 (63)	124 (50)	73 (51)		1291 (71)	132 (55)	90 (49)	73 (46)	
Frequently	523 (29)	72 (34)	129 (52)	83 (57)		550 (30)	60 (37)	126 (50)	71 (49)		520 (29)	107 (45)	93 (51)	87 (54)	
**Body mass index**					**					**					**
<30 kg/m^2	1531 (86)	175 (85)	199 (81)	109 (76)		1569 (86)	135 (84)	202 (83)	108 (76)		1551 (86)	190 (82)	149 (82)	124 (78)	
≥30 kg/m^2	241 (14)	31 (15)	46 (19)	35 (24)		251 (14)	25 (16)	42 (17)	35 (24)		243 (14)	42 (18)	33 (18)	35 (22)	

[a] *p*-values from the Pearson's Chi^2 tests: *$p \leq 0.05$, **$p \leq 0.01$, ***$p \leq 0.001$

Table S3. Descriptive statistics of childhood disadvantage and adult behavioral risk factors by adult psychological distress, measured by the subscales of the Depression Anxiety Stress Scales 21 (DASS-21), among men (n, %).

	Depressive symptoms					Anxiety symptoms					Stress symptoms				
Childhood disadvantage	**Normal**	**Mild**	**Moderate**	**Severe**	***p*** [a]	**Normal**	**Mild**	**Moderate**	**Severe**	***p*** [a]	**Normal**	**Mild**	**Moderate**	**Severe**	***p*** [a]
**Own serious/ long-term illness**					***					*					*
No	365 (95)	61 (86)	53 (83)	45 (96)		422 (94)	28 (88)	43 (91)	31 (82)		433 (94)	35 (90)	28 (80)	28 (90)	
Yes	19 (5)	10 (14)	11 (17)	2 (4)		27 (6)	4 (13)	4 (9)	7 (18)		28 (6)	4 (10)	7 (20)	3 (10)	
**Parental divorce**															
No	275 (71)	44 (62)	41 (62)	26 (55)		315 (70)	21 (64)	26 (54)	24 (63)		319 (68)	27 (69)	21 (58)	19 (61)	
Yes	113 (29)	27 (38)	25 (38)	21 (45)		138 (30)	12 (36)	22 (46)	14 (37)		147 (32)	12 (31)	15 (42)	12 (39)	
**Parental death**															
No	361 (94)	67 (94)	61 (95)	44 (96)		423 (94)	29 (91)	46 (98)	35 (95)		433 (94)	38 (97)	33 (94)	29 (97)	
Yes	24 (6)	4 (6)	3 (5)	2 (4)		27 (6)	3 (9)	1 (2)	2 (5)		29 (6)	1 (3)	2 (6)	1 (3)	
**Parental mental illness**															
No	343 (89)	62 (87)	51 (78)	37 (80)		397 (88)	28 (88)	37 (79)	31 (84)		408 (88)	32 (82)	29 (83)	24 (80)	
Yes	43 (11)	9 (13)	14 (22)	9 (20)		55 (12)	4 (13)	10 (21)	6 (16)		56 (12)	7 (18)	6 (17)	6 (20)	
**Parental alcohol problems**					*										
No	302 (78)	51 (72)	43 (66)	30 (61)		341 (75)	24 (73)	34 (71)	27 (71)		352 (76)	31 (78)	20 (57)	23 (72)	
Yes	85 (22)	20 (28)	22 (34)	19 (39)		112 (25)	9 (27)	14 (29)	11 (29)		113 (24)	9 (23)	15 (43)	9 (28)	
**Family economic hardship**					***										**
No	319 (82)	46 (65)	39 (59)	31 (67)		353 (78)	24 (73)	35 (73)	23 (62)		367 (78)	28 (72)	19 (54)	21 (70)	
Yes	70 (18)	25 (35)	27 (41)	15 (33)		101 (22)	9 (27)	13 (27)	14 (38)		101 (22)	11 (28)	16 (46)	9 (30)	
**Peer bullied**					***					***					***
No	295 (76)	57 (79)	32 (49)	23 (48)		339 (75)	25 (76)	27 (56)	16 (42)		353 (76)	23 (59)	18 (51)	13 (41)	
Yes	93 (24)	15 (21)	33 (51)	25 (52)		115 (25)	8 (24)	21 (44)	22 (58)		114 (24)	16 (41)	17 (49)	19 (59)	
**Parental education**										*					
High	240 (61)	38 (53)	34 (51)	24 (49)		272 (59)	24 (73)	23 (46)	17 (45)		283 (59)	21 (53)	18 (50)	14 (44)	
Low	156 (39)	34 (47)	33 (49)	25 (51)		191 (41)	9 (27)	27 (54)	21 (55)		193 (41)	19 (48)	18 (50)	18 (56)	
Behavioral factors															
**Fruit and vegetable consumption**															
Daily	254 (64)	40 (56)	40 (60)	26 (53)		295 (64)	15 (45)	27 (54)	23 (61)		297 (62)	21 (53)	23 (64)	19 (59)	
Non-daily	142 (36)	32 (44)	27 (40)	23 (47)		168 (36)	18 (55)	23 (46)	15 (39)		179 (38)	19 (48)	13 (36)	13 (41)	
**Leisure-time physical activity**					**					***					***
High/moderate	352 (90)	59 (84)	53 (79)	36 (73)		411 (90)	29 (88)	35 (70)	25 (66)		423 (90)	33 (83)	23 (64)	21 (66)	
Low	40 (10)	11 (16)	14 (21)	13 (27)		46 (10)	4 (12)	15 (30)	13 (34)		47 (10)	7 (18)	13 (36)	11 (34)	
**Alcohol use**															
No/moderately	242 (62)	45 (63)	33 (50)	30 (63)		288 (63)	14 (42)	25 (51)	23 (61)		283 (60)	29 (73)	20 (57)	18 (56)	
Excessively	150 (38)	27 (38)	33 (50)	18 (38)		170 (37)	19 (58)	24 (49)	15 (39)		188 (40)	11 (28)	15 (43)	14 (44)	

**Tobacco use**					***					*					
No/not nowadays	270 (68)	41 (57)	31 (46)	36 (75)		313 (67)	17 (53)	23 (46)	25 (66)		311 (65)	22 (55)	22 (61)	23 (72)	
Occasionally/daily	127 (32)	31 (43)	36 (54)	12 (25)		151 (33)	15 (47)	27 (54)	13 (34)		165 (35)	18 (45)	14 (39)	9 (28)	
**Sleep problems**					***					***					***
No/seldom	337 (85)	57 (79)	39 (58)	29 (59)		382 (82)	25 (76)	29 (58)	26 (68)		395 (83)	31 (78)	21 (58)	15 (47)	
Frequently	60 (15)	15 (21)	28 (42)	20 (41)		82 (18)	8 (24)	21 (42)	12 (32)		82 (17)	9 (23)	15 (42)	17 (53)	
**Body mass index**										***					
<30 kg/m^2	343 (87)	57 (79)	53 (79)	36 (75)		398 (86)	31 (94)	34 (68)	26 (70)		407 (86)	28 (70)	30 (83)	24 (77)	
≥30 kg/m^2	52 (13)	15 (21)	14 (21)	12 (25)		64 (14)	2 (6)	16 (32)	11 (30)		68 (14)	12 (30)	6 (17)	7 (23)	

[a] *p*-values from the Pearson's Chi^2 tests: *$p \leq 0.05$, **$p \leq 0.01$, ***$p \leq 0.001$

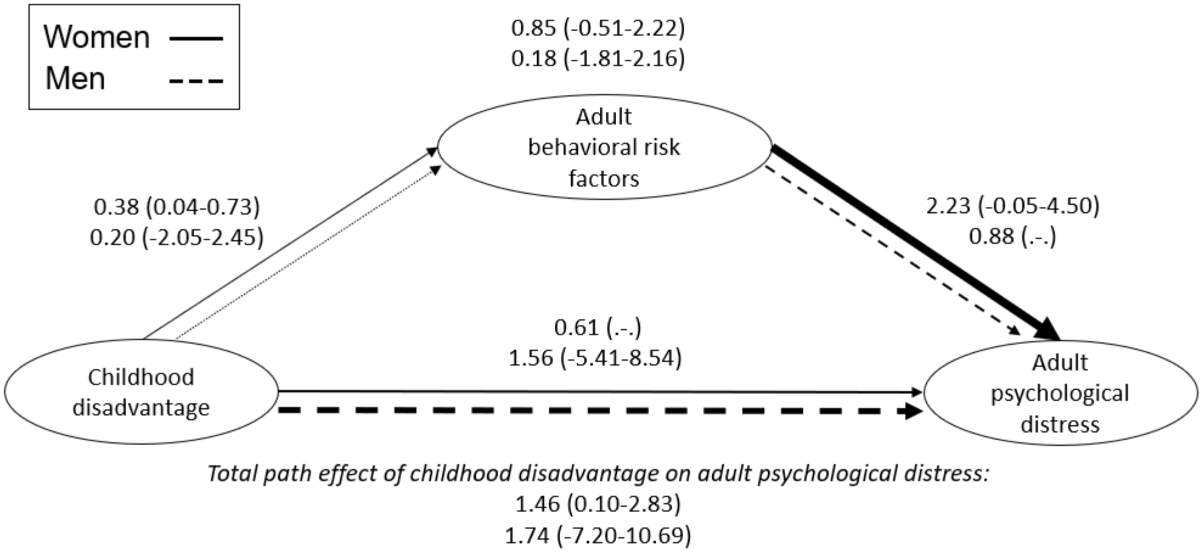

Figure S1. Generalized structural equation model of the pathways between the latent variables of childhood disadvantage (8 items), adult behavioral risk factors (5 items), and adult psychological distress (depressive, anxiety, and stress symptom subscales from the Depression Anxiety Depression Scale 21 (DASS-21)). The Helsinki Health Study cohort participants in Phases 1 (2017) and 2 (2022). The pathway effect of childhood disadvantage on adult behavioral risk factors is adjusted for age. Estimated path coefficients (β-values) and their 95% confidence intervals are shown: upper estimates and solid lines for women (n=2397) and lower estimates and dashed lines for men (n=586). Thicker lines indicate stronger path effects.

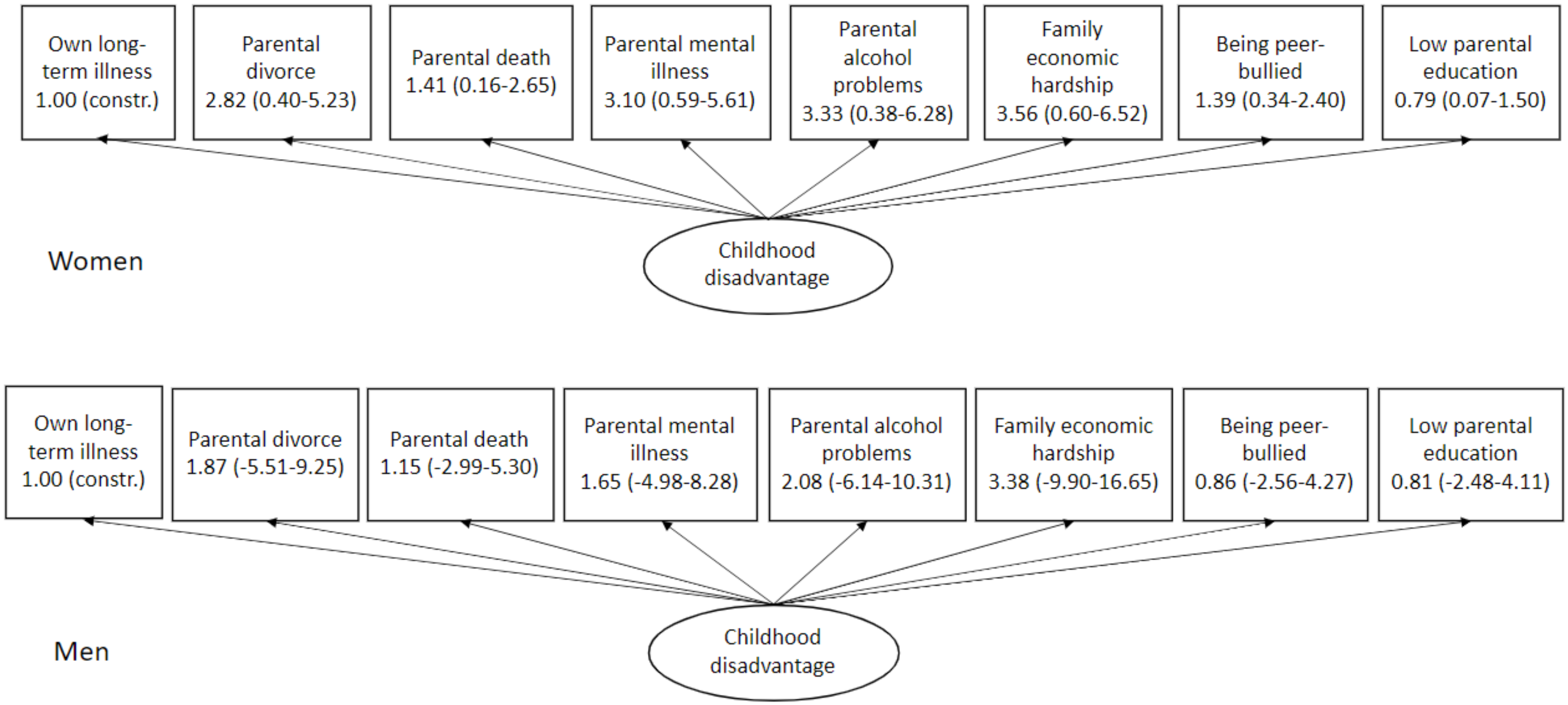

Figure S2. Estimated path coefficients (β-values) and their 95% confidence intervals for the latent variable of childhood disadvantage, consisting of 8 measures, among women (n=2397) and men (n=586). The Helsinki Health Study cohort participants in Phases 1 (2017) and 2 (2022). Estimates derived from the full generalized structural equation model (Figure S1) of the pathways between latent variables of childhood disadvantage, adult behavioral risk factors, and adult psychological distress. Circles = latent variables, boxes = observed variables.

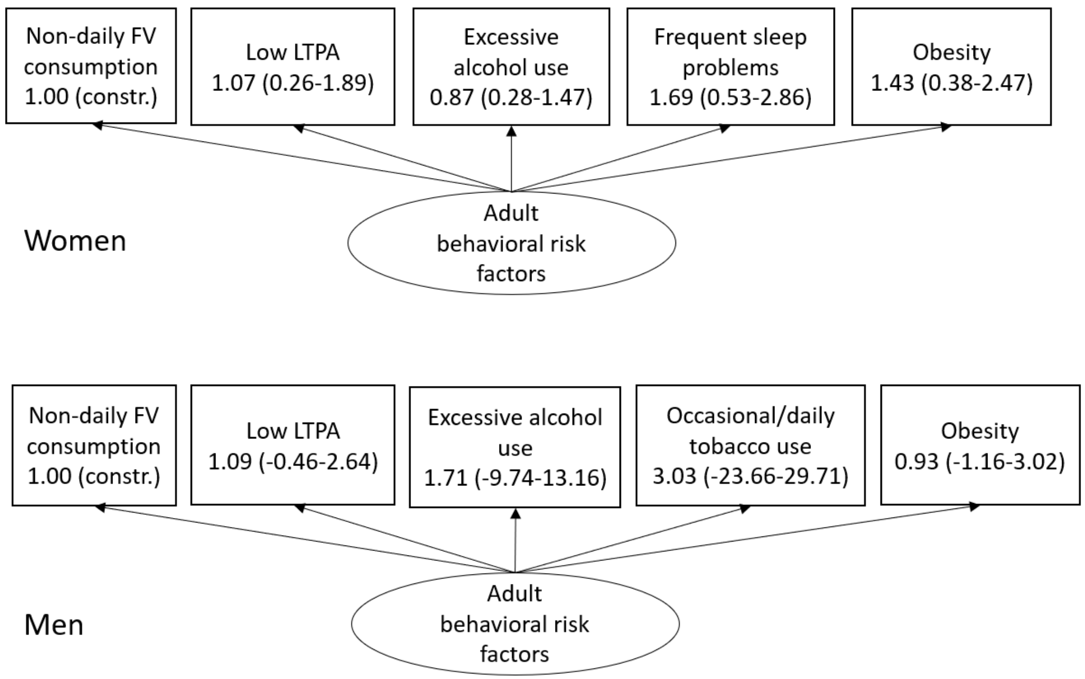

Figure S3. Estimated path coefficients (β-values) and their 95% confidence intervals for the latent variable of adult behavior risk factors, consisting of 5 measures, among women (n=2397) and men (n=586). The Helsinki Health Study cohort participants in Phases 1 (2017) and 2 (2022). Estimates derived from the full generalized structural equation models (Figure S1) of the pathways between childhood disadvantage, adult behavioral risk factors, and adult psychological distress. FV = fruit and vegetable, LTPA = leisure-time physical activity. Circles = latent variables, boxes = observed variables.

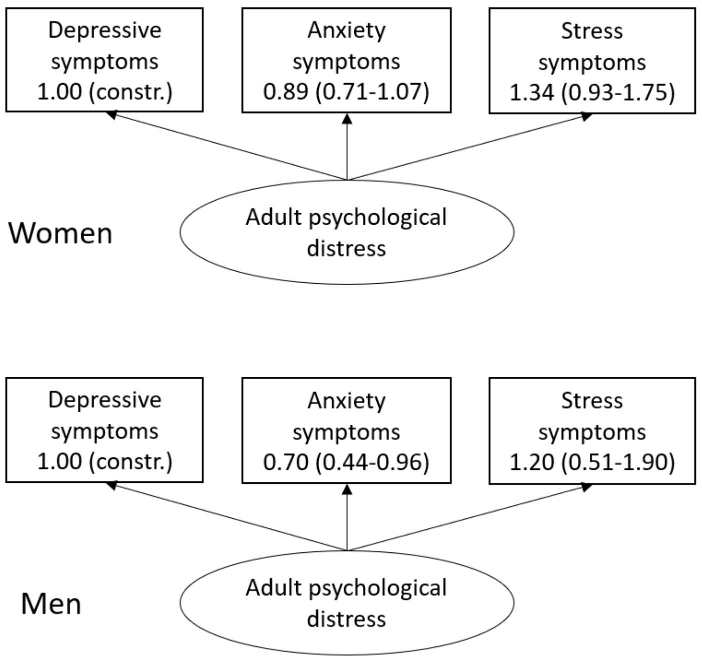

Figure S4. Estimated path coefficients (*β*-values) and their 95% confidence intervals for the latent variable of adult psychological distress, consisting of the three DASS-21 subscales, among women (n=2397) and men (n=586). The Helsinki Health Study cohort participants in Phases 1 (2017) and 2 (2022). Estimates derived from the full generalized structural equation models (Figure S3) of the pathways between childhood disadvantage, adult behavioral risk factors, and adult psychological distress. Circles = latent variables, boxes = observed variables.

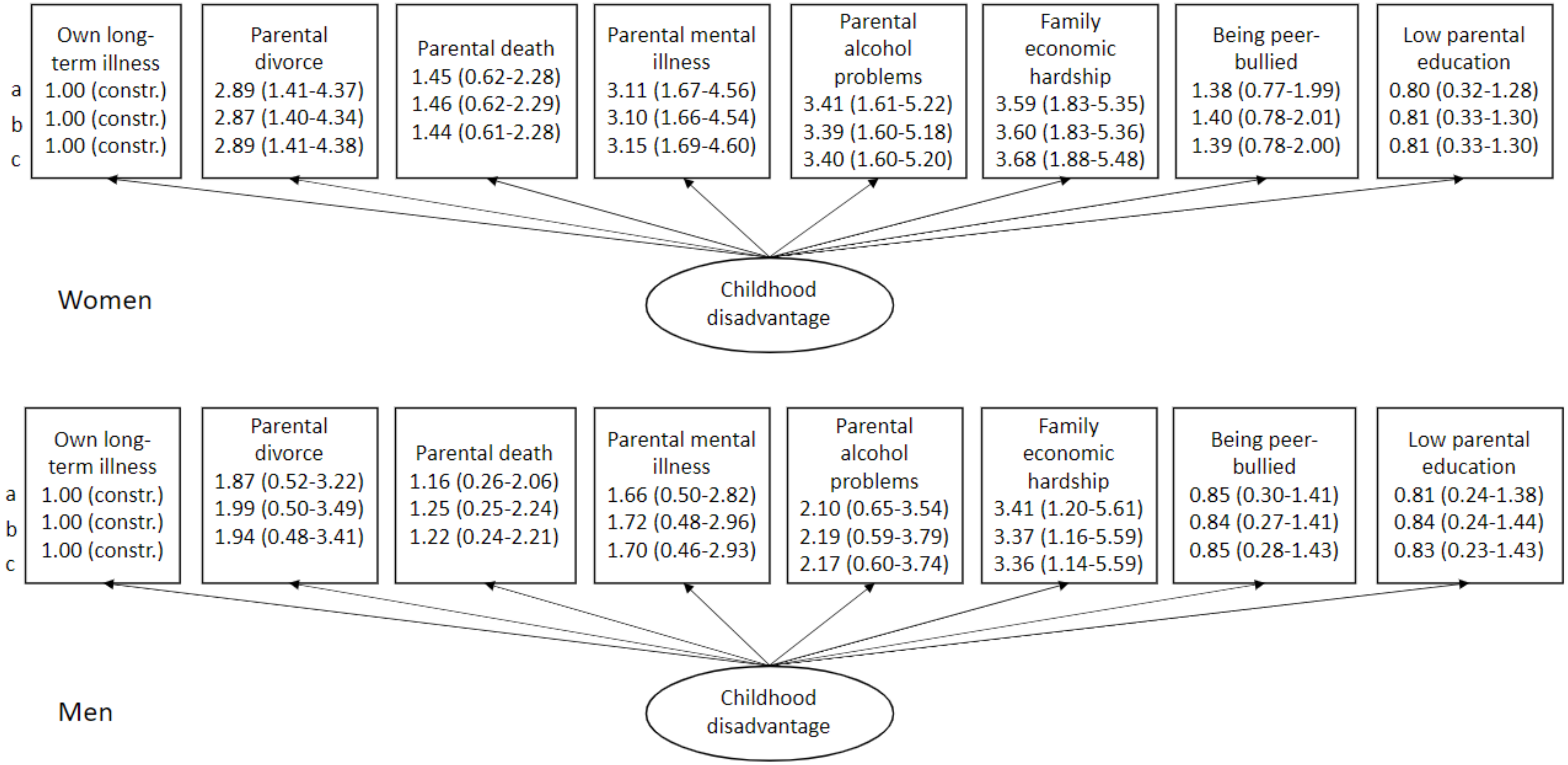

Figure S5. Estimated path coefficients (*β*-values) and their 95% confidence intervals for the latent variable of childhood disadvantage, consisting of 8 measures, among women (n=2397) and men (n=586). The Helsinki Health Study cohort participants in Phases 1 (2017) and 2 (2022). Estimates derived from the full generalized structural equation models (Figure 3) of the pathways between childhood disadvantage, adult behavioral risk factors, and adult psychological distress using the subscales of Depression Anxiety Stress Scales 21 (DASS-21) subscales: (a) depressive, (b) anxiety, and (c) stress symptoms. Circles = latent variables, boxes = observed variables.

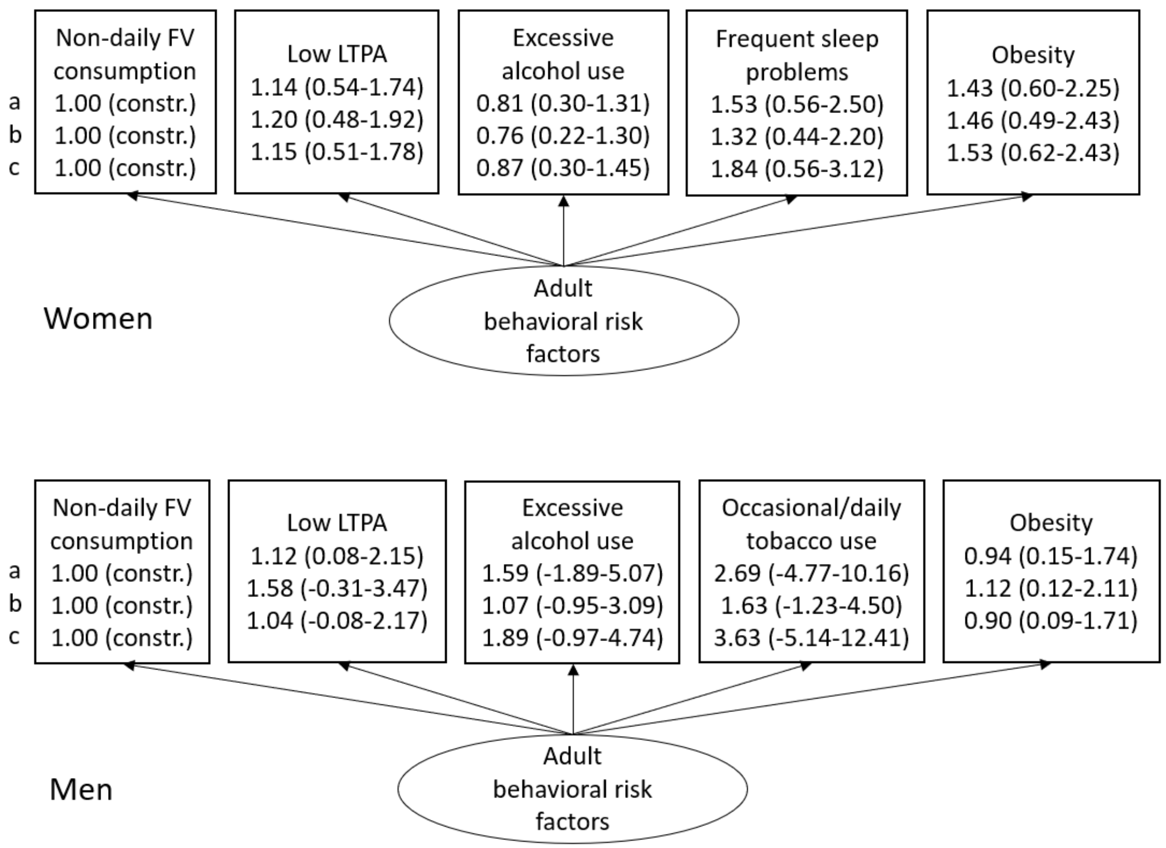

Figure S6. Estimated path coefficients (β-values) and their 95% confidence intervals for the latent variable of adult behavioral risk factors, consisting of 5 measures, among women (n=2397) and men (n=586). The Helsinki Health Study cohort participants in Phases 1 (2017) and 2 (2022). Estimates derived from the full generalized structural equation models (Figure 3) of the pathways between childhood disadvantage, adult behavioral risk factors, and adult psychological distress using the subscales of Depression Anxiety Stress Scales 21 (DASS-21): (a) depressive, (b) anxiety, and (c) stress symptoms. FV = fruit and vegetable, LTPA = leisure-time physical activity. Circles = latent variables, boxes = observed variables.
